# DIFFERENCES IN SEX-RELATED PERSONALITY TRAITS AND POLYPHARMACY AMONG OLDER ADULTS WITH DEPRESSIVE DISORDERS

**DOI:** 10.1093/geroni/igad104.3281

**Published:** 2023-12-21

**Authors:** Tsung-Yu Tsai, Tzu-Yun Wang, Yi-Hsin Wang, Kao Chin Chen, Huai-Hsuan Tseng, Ching-Ju Chiu

**Affiliations:** National Cheng Kung University Hospital, Tainan, Tainan, Taiwan (Republic of China); National Cheng Kung University Hospital, Tainan, Tainan, Taiwan (Republic of China); Tri-Service General Hospital, Taipei, Taipei, Taiwan (Republic of China); National Cheng Kung University Hospital, Tainan, Tainan, Taiwan (Republic of China); National Cheng Kung University Hospital, Tainan, Tainan, Taiwan (Republic of China); National Cheng Kung University, Tainan, Tainan, Taiwan (Republic of China)

## Abstract

This cross-sectional study aimed to explore the potential correlations between personality traits and the occurrence of polypharmacy (defined as the use of five or more medications) in older adults diagnosed with depressive disorders. In this study, 90 participants (30 males, 60 females) with an average age of 71.3±5.0 years were included. Personality traits were evaluated using the short version of the 50-item International Personality Item Pool, while medication usage data were retrieved from the National Health Insurance Pharma Cloud Database. Hierarchical regression models were employed for data analysis to examine the associations between personality traits and polypharmacy. The results showed that the mean number of medications taken was 10.9 ± 6.8 and that 84.4% of the participants satisfied the criterion for polypharmacy. Notably, low levels of openness significantly correlated with a higher number of medications taken (B=-0.38, p< 0.01). Gender-specific analyses demonstrated that low levels of conscientiousness (B=-0.07, p=0.01) and openness (B=-0.49, p=0.03) correlated with polypharmacy among males, whereas these personality traits displayed no significant correlations with polypharmacy in females (B=0.05, p=0.77, and B=-0.28, p=0.07, respectively). The findings underscore the elevated risk of polypharmacy among older adults diagnosed with depressive disorder. Additionally, the study highlights the necessity of considering gender differences in the interplay between personality traits and medication utilization patterns.

